# Lonely Points in Simplices

**DOI:** 10.1007/s00454-022-00428-2

**Published:** 2022-09-29

**Authors:** Maximilian Jaroschek, Manuel Kauers, Laura Kovács

**Affiliations:** 1 QAware Gmbh, Aschauer Straße 32, 81549 München, Germany; 2 Institute for Algebra, Johannes Kepler University Linz, Altenbergerstrasse 69, Linz, 4040 Austria; 3 Institute for Logics and Computation, TU Wien, Favoritenstrasse 9–10, Wien, 1040 Austria

**Keywords:** Integer points, Polytopes, Lattices, Discrete geometry, 52C07

## Abstract

Given a lattice $L\subseteq \mathbb Z^m$ and a subset $A\subseteq \mathbb R^m$, we say that a point in *A* is *lonely* if it is not equivalent modulo $L$ to another point of *A*. We are interested in identifying lonely points for specific choices of $L$ when *A* is a dilated standard simplex, and in conditions on $L$ which ensure that the number of lonely points is unbounded as the simplex dilation goes to infinity.

## Introduction

The geometric problem considered in this article arose from an attempt to construct an algorithm for simplifying so-called C-finite sequences. A sequence $(a_n)_{n=0}^\infty$ in the field $\mathbb C$ of complex numbers is called *C-finite* [11] if it satisfies a linear recurrence with constant coefficients^1^, i.e., if there are constants $c_0,\ldots ,c_r\in \mathbb C$, not all zero, such that

$$\begin{aligned} c_0a_n + c_1a_{n+1} + \cdots + c_ra_{n+r} = 0 \end{aligned}$$

for all $n\in \mathbb N$. A standard example is the sequence of Fibonacci numbers (take $c_0=c_1=1$ and $c_2=-1$). C-finite sequences and their properties are very well understood [7, 11, 12, 16, 17]. In particular, it is known that a sequence is C-finite if and only if it can be expressed as a linear combination of exponential terms with polynomial coefficients, i.e., if there are polynomials $p_1,\ldots ,p_m\in \mathbb C[x]$ and constants $\phi _1,\ldots ,\phi _m\in \mathbb C$, such that

$$\begin{aligned} a_n = p_1(n)\phi _1^n + \cdots + p_m(n)\phi _m^n \end{aligned}$$

for all $n\in \mathbb N$. We note that $\mathbb N$, as it is used in this paper, always contains 0. If the $\phi _i$ are pairwise distinct and all the $p_i$ are nonzero, then the order *r* of the corresponding recurrence turns out to be $m+\sum _{i=1}^m\deg (p_i)$.

One of the consequences of the characterization above is that the class of C-finite sequences is closed under addition and multiplication, i.e., when the sequences $(a_n)_{n=0}^\infty$ and $(b_n)_{n=0}^\infty$ are C-finite, then so are the sequences $(a_n+b_n)_{n=0}^\infty$ and $(a_nb_n)_{n=0}^\infty$. In particular, when we plug a C-finite sequence into a polynomial, the result is again a C-finite sequence. For example, since the sequence $(F_n)_{n=0}^\infty$ of Fibonacci-numbers is C-finite, so is the sequence $(5F_n^3-7F_n^2+9F_n-4)_{n=0}^\infty$ obtained by plugging $(F_n)_{n=0}^\infty$ into the polynomial $5x^3-7x^2+9x-4\in \mathbb C[x]$.

Given a C-finite sequence $(a_n)_{n=0}^\infty$, specified by a recurrence of order *r* and a set of initial values, we want to decide whether there is a polynomial $q\in \mathbb C[x]$ of positive degree such that the C-finite sequence $(q(a_n))_{n=0}^\infty$ satisfies a recurrence of order less than *r*. This problem is of interest because certain number-theoretic questions about C-finite sequences can at the moment only be answered when the recurrence order is not too large. In particular, the question whether for a given C-finite sequence $(a_n)_{n=0}^\infty$ there exists an index *n* such that $a_n=0$ is only known to be decidable for C-finite sequences satisfying recurrences of small order. See [14] for a detailed discussion of the state of the art. By using results of our paper to pass from $(a_n)_{n=0}^\infty$ to $(q(a_n))_{n=0}^\infty$, we hope to extend the scope of these algorithms and advance, for example, their use in applications of static analysis of computer systems. Static program analysis requires the synthesis of polynomials $q\in \mathbb C[x]$ corresponding to polynomial invariants $q(x) = 0$ among program variables *x*. These invariants in turn describe algebraic relations among C-finite sequences $(a_n)_{n=0}^\infty$ induced by the value distributions of program variables at arbitrary loop iterations $n\ge 0$. Moreover, one is interested in synthesizing a minimal set of polynomial invariants $q(x) = 0$, in particular polynomial invariants with small degrees enabling scalable approaches to static analysis. As such, results of our paper may potentially contribute to the full and efficient automation of polynomial invariant generation within software verification. See for example [9, 10] for further details.

The construction of an algorithm for finding $q\in \mathbb C[x]$, such that $(q(a_n))_{n=0}^\infty$ yields a C-finite sequence of lower order than *a*, has led us to the following geometric problem. Let $S\subseteq \mathbb R^m$ be the standard simplex, i.e., the convex hull of 0 and the unit vectors $e_1,\ldots ,e_m\in \mathbb R^m$. Moreover, let $L\subseteq \mathbb Z^m$ be a lattice, i.e., an additive subgroup of $\mathbb Z^m$. Two points $u,v\in \mathbb R^m$ are called equivalent modulo $L$ if we have $u-v\in L$. We consider the integer points in a dilation $dS$ of $S$, for some $d>0$. A point $u\in dS\cap \mathbb Z^m$ is called *lonely* if there does not exist any other point $v\in dS\cap \mathbb Z^m$ such that $u-v\in L$. Equivalently, *u* is lonely if it is covered exactly once in the lattice arrangement $L+dS$. In this paper, we are interested in describing properties of these lonely points.

In Sect. 2, we will give some more details on how the original problem about C-finite sequences leads to the consideration of lonely points. This material is provided only as background information and not strictly needed for the rest of the paper. In Sect. 3, we summarize basic definitions and facts about cones, simplices, and lattices, and fix the notation we use. In Sect. 4 we present algorithms that for a given lattice $L$ and a given *d* determine all the lonely points, and recognize whether the number is unbounded as *d* goes to infinity. Finally, in Sect. 5 we derive a sufficient condition on the lattice that guarantee that the number of lonely points is unbounded.

## Ansatz and Exponent Lattice

Consider a C-finite sequence $(a_n)_{n=0}^\infty$ which satisfies a recurrence of order *r*. We want to know whether there is a polynomial $q\in \mathbb C[x]\setminus \mathbb C$ such that $(q(a_n))_{n=0}^\infty$ satisfies a recurrence of lower order. If we have an upper bound *d* on the degree of *q*, then this question can be answered as follows:

1. Compute $p_1,\ldots ,p_m\in \mathbb C[x]$ and $\phi _1,\ldots ,\phi _m\in \mathbb C$ such that $a_n = p_1(n)\phi _1^n + \cdots + p_m(n)\phi _m^n$ for all $n\in \mathbb N$ (see [11] for how to do this).
2. Make an ansatz $q=q_0+q_1x+\cdots +q_dx^d$ with undetermined coefficients $q_0,\ldots ,q_d$, plug the closed form representation of step 1 into *q*.
3. Write the resulting expression in the form $u_1\psi _1^n + \cdots + u_\ell \psi _\ell ^n$ where the $\psi _i\in \mathbb C$ are pairwise distinct and the $u_i$ are polynomials in *n* whose coefficients are $\mathbb C$-linear combinations of the unknowns $q_0,\ldots ,q_d$.
4. For every subset $I\subseteq \{1,\ldots ,\ell \}$ such that $|\{1,\ldots ,\ell \}\setminus I|=r-1$, equate the coefficients with respect to *n* in all the $u_i$ belonging to some $\psi _i$ with $i\in I$ to zero and solve the resulting linear system for the unknowns $q_0,\ldots ,q_d$. If the solution space contains a vector $(q_0,\ldots ,q_d)$ in which not only $q_0$ is nonzero, return the corresponding polynomial $q_0+q_1x+\cdots +q_dx^d$. Otherwise, try the next *I*.
5. When no subset *I* yields a solution, return “there is no such *q*”.

### Example 2.1

1. The C-finite sequence $(a_n)_{n=0}^\infty$ with $a_n=1+2^n+2^{-n}$ satisfies a recurrence of order 3 and no lower order recurrence. With $d=2$, the algorithm sketched above finds the polynomial $q(x)=x^2-2x-1$. Indeed, $q(a_n)=4^n+4^{-n}$ satisfies a recurrence of order 2.
2. The C-finite sequence $(a_n)_{n=0}^\infty$ with $a_n=1+3^n+3^{2 n}+2\cdot 3^{3 n}-2\cdot 3^{4 n}$ satisfies a recurrence of order 5 and no lower order recurrence. For this input, the algorithm finds the polynomial $q(x)=x^2-3x+2$, and indeed, $q(a_n)=-3^n+7\cdot 3^{4 n}-8\cdot 3^{7 n}+4\cdot 3^{8n}$ satisfies a recurrence of order 4. Similar examples can be constructed using polynomials with sparse powers. Such polynomials have been studied for example in [5].
3. The C-finite sequence $(a_n)_{n=0}^\infty$ with $a_n=1+2^n-2^{-n}$ satisfies a recurrence of order 3, and with the algorithm sketched above we can show that there is no polynomial *q* of degree $d\le 5$ such that $q(a_n)$ satisfies a recurrence of order 2.

When we have checked the existence of a polynomial *q* for a specific degree *d* and found that no such polynomial exists, we can try again with a larger choice of *d*. It would be good to know when we can stop: starting from the recurrence of $(a_n)_{n=0}^\infty$, can we determine a finite bound on the degree of the polynomials *q* that may lead to lower order recurrences?

In order to see from where such a bound could emerge, restrict the search to polynomials *q* with $q_d=1$. Observe what happens in step 2 of the procedure sketched above. Plugging the expression $p_1(n)\phi _1^n + \cdots + p_m(n)\phi _m^n$ into the ansatz for *q* produces

1 $$\begin{aligned} \begin{aligned} q_0 + q_1 \sum _{i=1}^m p_i(n)\phi _i^n&+ q_2 \sum _{i,j=1}^n p_i(n)p_j(n)(\phi _i\phi _j)^n \\&+ \cdots + \sum _{i_1,\ldots ,i_d=1}^n \,\prod _{j=1}^dp_{i_j}(n)\left( \,\prod _{j=1}^d\phi _{i_j}\right) ^{\!n}, \end{aligned} \end{aligned}$$

so the $\psi _i$’s appearing in step 3 are precisely the products $\phi _1^{v_1}\ldots \phi _m^{v_m}$ with $v_1+\cdots +v_m\le d$. If these products are all distinct, then there is no way for the above expression to vanish identically. More generally, a necessary condition for the above expression to vanish identically for some choice of $q_0,\ldots ,q_{d-1}$, not all zero, is that a sufficient amount of cancellation takes place among the various exponential sequences $((\phi _1^{v_1}\ldots \phi _m^{v_m})^n)_{n=0}^\infty$.

This leads to the consideration of the so-called *exponent lattice*

$$\begin{aligned} L = \bigl \{(v_1,\ldots ,v_m)\in \mathbb Z^m : \phi _1^{v_1}\ldots \phi _m^{v_m}=1\bigr \}\subseteq \mathbb Z^m, \end{aligned}$$

which also plays an important role for determining the algebraic relations among C-finite sequences [13]. For example, for the Fibonacci numbers, where we have $\phi _1=(1+\sqrt{5})/2$ and $\phi _2=(1-\sqrt{5})/2$, the exponent lattice is generated by (2, 2).

A term $(\phi _1^{v_1}\ldots \phi _m^{v_m})^n$ appearing in (1) cannot be canceled unless there is some other point $(\tilde{v}_1,\ldots ,\tilde{v}_m)\in \mathbb N^m$ with $\tilde{v}_1+\cdots +\tilde{v}_m\le d$ and $(v_1-\tilde{v}_1,\ldots ,v_m-\tilde{v}_m)\in L$. If *d* is such that *r* or more of the terms have no partner for cancellation, then it is clear that there is no solution *q* of degree *d*. Moreover, if $L$ is such that the number of terms without partner tends to infinity as *d* increases, then there is a finite bound on the degree that a solution *q* may have.

## Lattices and Cones

We start by recalling some basic concepts from discrete geometry. Further background can be found in [2], for example.

### Definition 3.1

(lattices)   A set $L\subset \mathbb Z^m$ is called a *lattice* if it contains the origin and for all $u,v\in L$ and all $\alpha ,\beta \in \mathbb Z$ also $\alpha u+\beta v$ is an element of $L$. For vectors $\ell _1,\ldots ,\ell _k\in \mathbb Z^m$ we write $\langle \ell _1,\ldots ,\ell _k\rangle$ for the smallest lattice containing $\ell _1,\ldots ,\ell _k$, which we call generators of the lattice. The dimension $\dim (L)$ of a lattice is defined as the dimension of the $\mathbb R$-vector space it generates.

We always view a lattice $L\subseteq \mathbb Z^m$ as a set of points in the ambient space $\mathbb R^m$, spanned by the unit vectors $e_1,\ldots ,e_m$. In addition, it will be convenient to let $e_0$ be the zero vector. Note that we allow the dimension $\dim (L)$ of a lattice to be smaller than the dimension *m* of its ambient space.

### Example 3.1

**Fig. 1:**
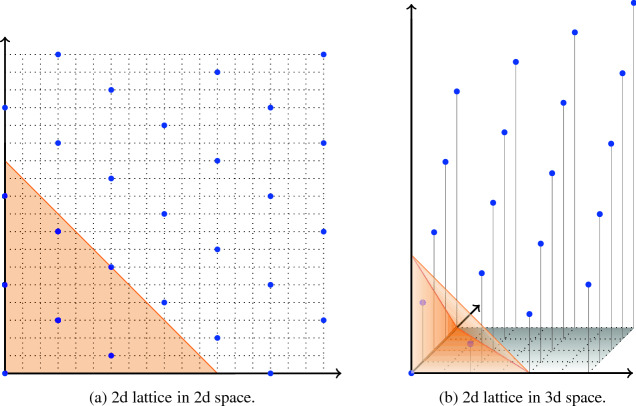
Lattices in the positive orthant. The orange areas mark the dilated simplices $12S$ and $4S$ respectively

The vectors (3, 3) and (6, 1) span a lattice in $\mathbb R^2$ of dimension 2. Some points in the lattice in the positive quadrant are depicted in Fig. 1a. The 2-dimensional lattice spanned by the vectors (2, 1, 0) and (0, 2, 1) in $\mathbb R^3$ is illustrated in Fig. 1.

### Definition 3.2

 (standard simplex)  The *standard simplex* $S$ in $\mathbb R^m$ is the convex hull of the points $e_0,\ldots ,e_m$. For $d\in \mathbb N$, the *d*-dilation $dS$ of $S$ is the convex hull of the points $d e_0,\ldots ,d e_m$.

We are interested in the integer points of a dilated simplex $dS\subseteq \mathbb R^m$. Obviously, this set consists of all points $(v_1,\ldots ,v_m)$ in $\mathbb Z^m$ with $v_1,\ldots ,v_m\ge 0$ and $v_1+\cdots +v_m\le d$. We can also describe it as an intersection of translated cones.

### Definition 3.3

 (cones)  A set $C\subseteq \mathbb Z^m$ is called a (*discrete*) *cone* if $C$ contains the origin and we have that for all $u,v\in C$ and for all $\alpha ,\beta \in \mathbb N$, the linear combination $\alpha u+\beta v$ is also an element of $C$. For vectors $c_1,\ldots ,c_n\in \mathbb Z^m$ we write $[c_1,\ldots ,c_n]$ for the smallest cone containing $c_1,\ldots ,c_n$, which we call generators of the cone. For a nonzero $c\in C$, [*c*] is called an *edge* of $C$ if there exists a hyperplane $H\subset \mathbb R^m$ with $H\cap C\subseteq [c]$. We call edges of the form $[e_i]$ or $[-e_i]$, $i\in \{1,\dots ,m\}$, *straight*, while all other edges are called *slanted*. For $i\in \{0,\ldots ,m\}$, we define the *i*th *corner cone* $C_i$ of the standard simplex as $[e_0-e_i,e_1-e_i\ldots ,e_m-e_i]\subseteq \mathbb Z^m$.

Subsequently, we will only be concerned with finitely generated cones. We can therefore assume that a cone $C$ is always given as a finite set of points $c_i$, such that for each *i*, $[c_i]$ is an edge of $C$, and for $j\ne i$ we have $[c_i]\ne [c_j]$.

**Fig. 2:**
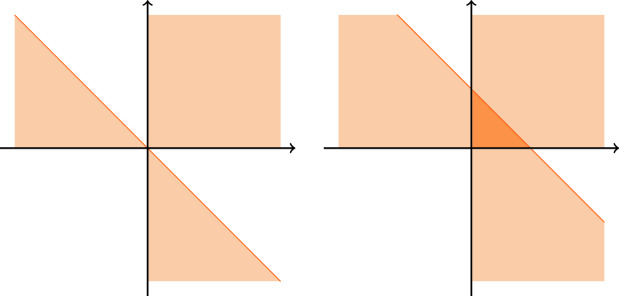
Corner cones of the standard simplex and the intersection of the translated cones in $\mathbb R^2$

The standard simplex in $\mathbb R^m$ has $m+1$ distinct corner cones $C_0,\ldots ,C_m$, and the set of all integer points in $dS$, $d\in \mathbb N$, is equal to the intersection $\bigcap _{i=0}^{m} (C_i-de_i)$, as illustrated for dimension 2 in Fig. 2.

As we outlined in the earlier sections, we look for integer points in $dS$ that are not connected to any other integer points in $dS$ via a given lattice $L$. The next definition formalizes this idea not only for simplices but general subsets of $\mathbb R^m$.

### Definition 3.4

(lonely points) Let $L\subseteq \mathbb Z^m$ be a lattice. We define the equivalence relation $\sim$ on $\mathbb Z^m$ as $u\sim v:\Leftrightarrow u-v\in L$. Let *A* be an arbitrary subset of $\mathbb R^m$. A point $v\in A$ is called *lonely* (with respect to $L$), if $v\in \mathbb Z^m$ and there is no $\tilde{v}\in (A\cap \mathbb Z^m)\setminus \{v\}$ such that $\tilde{v}- v\in L$. We write ${\text {lonely}}_{L}(A)$ for the set of lonely points in *A* and $\#{\text {lonely}}_{L}(A)\in \mathbb N\cup \{\infty \}$ for the number of lonely points in *A*.

### Example 3.2

We give two examples of lattices where the number of lonely points in *dS* does not grow indefinitely with *d*.

**Fig. 3:**
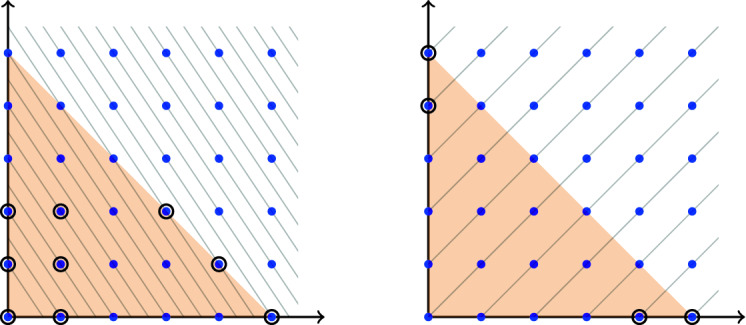
Illustration of Example 3.2. Lonely points are encircled

(i) For $$L=\bigl \langle \left( {\begin{array}{c}2\\ -3\end{array}}\right) \bigr \rangle \subseteq \mathbb Z^2$$ there are nine lonely points in all $dS$ for all $d\ge 4$ (Fig. 3, left). Note that three of them depend on *d* while the six others are identical for every *d*.
(ii) For $$L=\bigl \langle \left( {\begin{array}{c}1\\ 1\end{array}}\right) \bigr \rangle \subseteq \mathbb Z^2$$ there are four lonely points in all $dS$ for all $d\ge 2$ (Fig. 3, right). In this case, all four points vary with *d*.

It is easy to show that in $\mathbb Z^2$ there is no lattice (other than $\{0\}$) such that the number of lonely points in *dS* grows indefinitely with *d*.

### Example 3.3

Let $L\subseteq \mathbb Z^4$ be the lattice generated by the vectors $(2,0,-1,0)$ and $(1,1,0,-1)$. Then there are infinitely many lonely points in any corner cone. For example, for each $i=0,\ldots ,4$, all vectors of the form $(0,n,0,0)-de_i$ with $d\ge n\ge 0$ are lonely in $C_i$.

Our goal is to count the lonely points in a dilated simplex. As we will use the translated corner cones to characterize the points inside of a dilated simplex, we want to make sure that lonely points stay lonely after any translation.

### Lemma 3.1

Let $L\subset \mathbb Z^m$ be a lattice and let $v\in A\subseteq \mathbb R^m$. If $v\in {\text {lonely}}_{L}(A)$, then $v+t\in {\text {lonely}}_{L}(A+t)$ for any $t\in \mathbb Z^m$.

### Proof

Suppose $v+t\notin {\text {lonely}}_{L}(A+t)$. Then there exists a $\tilde{v}\in A$ such that $(v+t)\sim (\tilde{v}+t)$. It follows that $v-\tilde{v} = (v+t) - (\tilde{v} +t)\in L$, so $v\sim \tilde{v}$. $\square$

## Counting and Identifying Lonely Points

In this section we develop algorithms for deciding whether in a given setting the number of lonely points is finite or infinite, as well as an algorithm which in the finite case determines how many lonely points there are. First we characterize loneliness of points in cones, and then we relate the loneliness of points in a dilated simplex $dS$ to the loneliness of points in its corner cones.

### Lemma 4.1

Let $L\subseteq \mathbb Z^m$ be a lattice and $C\subseteq \mathbb Z^m$ be a cone.

(i) If $C$ has any lonely points, then 0 is one of them.
(ii) $C$ has lonely points if and only if $L\cap C=\{0\}$.
(iii) If $u\in C$ is not lonely, then also $u+v$ is not lonely for any $v\in C$.

### Proof

(i) If 0 is not lonely, it is equivalent to some other point of $C$, say to $u\ne 0$. Then $u=u-0\in L$. Let *v* be an arbitrary element of $C$. Since $u\in C$, we have $v+u\in C$, and since *v* and $v+u$ are equivalent, *v* is not lonely.
(ii) If $C$ has lonely points, then, by the previous item, 0 is one of them, hence $L\cap C=\{0\}$. For the other direction, if $L\cap C=\{0\}$, then 0 is lonely.
(iii) If *u* is not lonely, then there exists $\tilde{u}\in C\setminus \{u\}$ with $u\sim \tilde{u}$. Then also $u+v\sim \tilde{u}+v$, and since $\tilde{u}+v$ is in $C$ and different from $u+v$, the claim follows.

$$\square $$

### Proposition 4.1

Let $L\subseteq \mathbb Z^m$ be a lattice and $C=[c_1,\ldots ,c_n]\subseteq \mathbb Z^m$ be a cone.

(i) If $C$ has infinitely many lonely points, then there is an $i\in \{1,\ldots ,n\}$ such that all points in $[c_i]$ are lonely in $C$.
(ii) Let $i\in \{1,\ldots ,n\}$. Then all points in $[c_i]$ are lonely in $C$ if and only if $L\cap C=\{0\}$ and $(L+\langle c_i\rangle )\cap C=[c_i]$.

### Proof

(i) Suppose to the contrary all edges $[c_i]$ contain a nonlonely point, say $\alpha _1c_1,\dots ,\alpha _nc_n$ are not lonely for certain positive integers $\alpha _1,\dots ,\alpha _n$. By part  of Lemma 4.1, all points $\beta _1c_1+\cdots +\beta _nc_n$ with $\beta _1\ge \alpha _1,\dots ,\beta _n\ge \alpha _n$ are not lonely. Thus there remain only finitely many candidates for lonely points.
(ii) “$\Rightarrow$” If all points in $[c_i]$ are lonely, then $C$ has lonely points, so $L\cap C=\{0\}$ by of Lemma 4.1. It remains to shows that $(L+\langle c_i\rangle )\cap C=[c_i]$. The direction “$\supseteq$” is clear. To show “$\subseteq$”, let $v\in (L+\langle c_i\rangle )\cap C$, say $v=\ell +\alpha c_i\in C$ for some nonzero $\ell \in L$ and $\alpha \in \mathbb Z$. If $\alpha > 0$, then $v\sim \alpha c_i$, in contradiction to the loneliness of $\alpha c_i$. Otherwise, for $\alpha \le 0$, we have $\ell =v+(-\alpha )c_i\in C$, a contradiction to $L\cap C=\{0\}$. “$\Leftarrow$” Assume $u=\alpha _ic_i$ is not lonely, say $u\sim v$ for some $v\in C\setminus \{u\}$. Then $u-v\in L$ implies $v\in L+\langle c_i\rangle$, so $v\in [c_i]$, say $v=\beta _ic_i$ for some $\beta \in \mathbb N\setminus \{\alpha _i\}$. But then $0\ne {\text {sgn}}(\beta _i-\alpha _i)(u-v)\in L\cap C=\{0\}$, a contradiction.

$$\square $$

The conditions of Proposition 4.1 give rise to the following algorithm for deciding whether a cone contains infinitely many lonely points. 
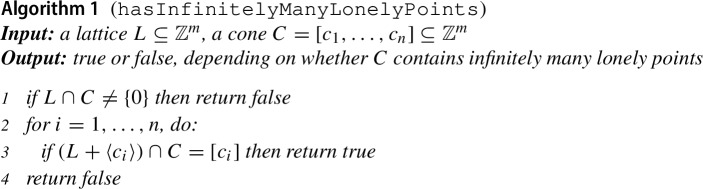


The tests in lines 1 and 3 can be performed using integer linear programming [15]. If $L=\langle \ell _1,\dots ,\ell _k\rangle =[\ell _1,\dots ,\ell _k,-\ell _1,\dots ,-\ell _k]$, we can find nonnegative integers $\alpha _1,\dots ,\alpha _k,\alpha _{-1},\dots ,\alpha _{-k},\beta _1,\dots ,\beta _n$ such that

$$\begin{aligned} (\alpha _1-\alpha _{-1})\ell _1+\dots +(\alpha _k-\alpha _{-k})\ell _k=\beta _1c_1+\dots +\beta _n c_n \end{aligned}$$

and such that $\beta _1+\cdots +\beta _n$ is maximized. We have $L\cap C=\{0\}$ if and only if the optimal solution is $\beta _1=\ldots =\beta _n=0$.

Similarly, in order to check whether $(L+\langle c_i\rangle )\cap C=[c_i]$, we can find nonnegative integers $\alpha _1,\dots ,\alpha _k,\alpha _{-1},\dots ,\alpha _{-k},\gamma _1,\gamma _{-1},\beta _1,\dots ,\beta _n$ such that

$$\begin{aligned} (\alpha _1-\alpha _{-1})\ell _1+\dots +(\alpha _k-\alpha _{-k})\ell _k+(\gamma _1-\gamma _{-1})c_i=\beta _1c_1+\dots +\beta _n c_n \end{aligned}$$

and $\beta _1+\cdots +\beta _{i-1}+\beta _{i+1}+\cdots +\beta _n$ is maximized. If the intersection $[c_i]\cap [c_1,\dots ,c_{i-1},c_{i+1},\dots ,c_n]$ only contains 0, then $(L+\langle c_i\rangle )\cap C$ is contained in $[c_i]$ if and only if the optimal solution is $\beta _1=\ldots =\beta _n=0$. In our setting, we can always assume that $c_1,\dots ,c_n$ are linearly independent over $\mathbb Q$, and in this case, the condition $[c_i]\cap [c_1,\dots ,c_{i-1},c_{i+1},\dots ,c_n]=\{0\}$ is always satisfied.

When there are only finitely many lonely points, we can next determine how many there are. Part  of Lemma 4.1 says that when some $v\in C$ is not lonely, then no point in the translated cone $v+C$ is lonely either. It follows from Dickson’s lemma ([3], see also Lemma 4 of [1]) that the set of nonlonely points in $C$ is in fact a finite union of such translated cones $v+C$, quite similar to the leading-term ideals in Gröbner basis theory [3, 4, 6]. Inspired by the FGLM-algorithm from that theory [6, 8], we arrive at the following algorithm for counting the number of lonely points in a cone. 
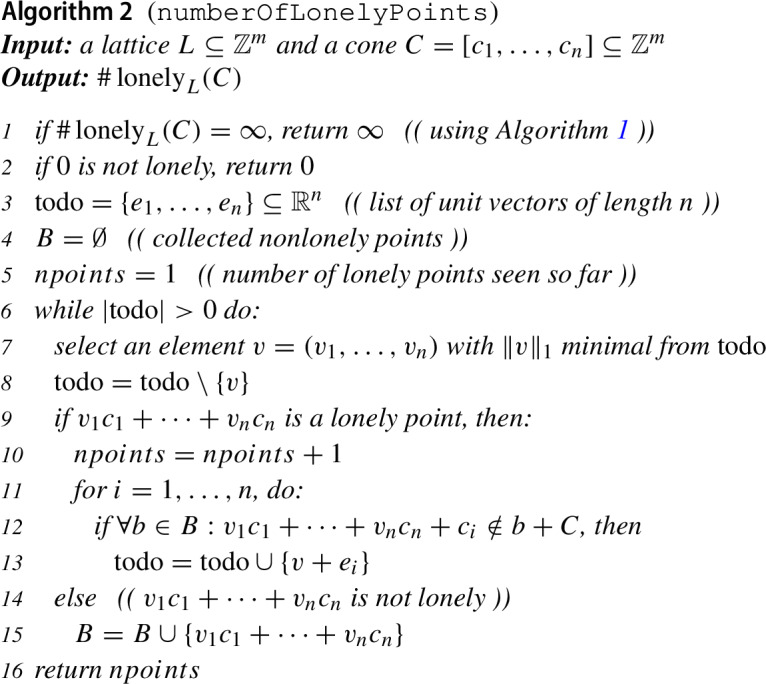


Three aspects need to be discussed in order to justify this algorithm: (1) that all indicated operations can be performed algorithmically, (2) that it returns the correct output, and (3) that it terminates for every input. Concerning the first point, the only questionable steps are the checks in steps 2 and 9 whether a given point is lonely. In order for *v* to be not lonely, there must be integers $\alpha _1,\dots ,\alpha _k$, not all zero, such that $v+\alpha _1\ell _1+\cdots +\alpha _k \ell _k$ also belongs to $C$, where $\ell _1,\dots ,\ell _k$ are generators of $L$. Whether such integers exist can be determined with integer linear programming [15].

For the correctness, observe first that the output *npoints* is a lower bound on the number of lonely points, because the counter is only incremented when we have found a new lonely point. Since we always consider the candidate of least 1-norm and in line 13 always add elements of larger 1-norm to the todo-list, it is excluded that we count the same point more than once. In order to see that the output is also an upper bound, observe that of Lemma 4.1 implies that when *b* is not lonely, then all the points in $b+C$ are not lonely either, so it is fair to exclude them from consideration in step 12. Since all other points will be considered, there is no danger of undercounting. This establishes the correctness.

Finally, for justifying the termination, observe that the number of iterations of the main loop is bounded by the number of lonely points plus the number of points that are not lonely but also not contained in a translated cone $b+C$ where *b* is a nonlonely point discovered earlier. By line 1, the number of lonely points is finite when the algorithm reaches the main loop, and we have already argued above that the number of nonlonely points not contained in a translated cone rooted at an earlier discovered nonlonely point is finite as well.

### Example 4.1

Consider the lattice $$L=\bigl \langle \left( {\begin{array}{c}2\\ -3\end{array}}\right) \bigr \rangle \subseteq \mathbb Z^2$$ and the cone $C=[e_1,e_2]\subseteq \mathbb Z^2$. This cone is the corner cone $C_0$ in the situation considered in Example 3.2 (i) and depicted in Fig. 3. Algorithm 2 identifies the lonely points of $C$ as follows.

**Table:** 

iteration	*v*	todo	*B*	*npoints*	comment
0		$$\bigl \{\left( {\begin{array}{c}1\\ 0\end{array}}\right) ,\left( {\begin{array}{c}0\\ 1\end{array}}\right) \bigr \}$$	$\emptyset$	1	initialization
1	$$\left( {\begin{array}{c}1\\ 0\end{array}}\right) $$	$$\bigl \{\left( {\begin{array}{c}2\\ 0\end{array}}\right) ,\left( {\begin{array}{c}1\\ 1\end{array}}\right) ,\left( {\begin{array}{c}0\\ 1\end{array}}\right) \bigr \}$$	$\emptyset$	2	*v* is lonely
2	$$\left( {\begin{array}{c}0\\ 1\end{array}}\right) $$	$$\bigl \{\left( {\begin{array}{c}2\\ 0\end{array}}\right) ,\left( {\begin{array}{c}1\\ 1\end{array}}\right) ,\left( {\begin{array}{c}0\\ 2\end{array}}\right) \bigr \}$$	$\emptyset$	3	*v* is lonely
3	$$\left( {\begin{array}{c}2\\ 0\end{array}}\right) $$	$$\bigl \{\left( {\begin{array}{c}1\\ 1\end{array}}\right) ,\left( {\begin{array}{c}0\\ 2\end{array}}\right) \bigr \}$$	$$\bigl \{\left( {\begin{array}{c}2\\ 0\end{array}}\right) \bigr \}$$	3	*v* is not lonely
4	$$\left( {\begin{array}{c}1\\ 1\end{array}}\right) $$	$$\bigl \{\left( {\begin{array}{c}1\\ 2\end{array}}\right) ,\left( {\begin{array}{c}0\\ 2\end{array}}\right) \bigr \}$$	$$\bigl \{\left( {\begin{array}{c}2\\ 0\end{array}}\right) \bigr \}$$	4	*v* is lonely
5	$$\left( {\begin{array}{c}0\\ 2\end{array}}\right) $$	$$\bigl \{\left( {\begin{array}{c}1\\ 2\end{array}}\right) ,\left( {\begin{array}{c}0\\ 3\end{array}}\right) \bigr \}$$	$$\bigl \{\left( {\begin{array}{c}2\\ 0\end{array}}\right) \bigr \}$$	5	*v* is lonely
6	$$\left( {\begin{array}{c}1\\ 2\end{array}}\right) $$	$$\bigl \{\left( {\begin{array}{c}1\\ 3\end{array}}\right) ,\left( {\begin{array}{c}0\\ 3\end{array}}\right) \bigr \}$$	$$\bigl \{\left( {\begin{array}{c}2\\ 0\end{array}}\right) \bigr \}$$	6	*v* is lonely
7	$$\left( {\begin{array}{c}0\\ 3\end{array}}\right) $$	$$\bigl \{\left( {\begin{array}{c}1\\ 3\end{array}}\right) \}$$	$$\{\left( {\begin{array}{c}2\\ 0\end{array}}\right) ,\left( {\begin{array}{c}0\\ 3\end{array}}\right) \bigr \}$$	6	*v* is not lonely
8	$$\left( {\begin{array}{c}1\\ 3\end{array}}\right) $$	$\emptyset$	$$\bigl \{\left( {\begin{array}{c}2\\ 0\end{array}}\right) ,\left( {\begin{array}{c}0\\ 3\end{array}}\right) ,\left( {\begin{array}{c}1\\ 3\end{array}}\right) \bigr \}$$	6	*v* is not lonely

The next proposition connects the lonely points in a simplex to the lonely points in its corner cones.

### Proposition 4.2

Let $L\subseteq \mathbb Z^m$ be a lattice and let $S\subseteq \mathbb R^m$ be the standard simplex.

(i) A corner $de_i$ of $dS$ is lonely for all sufficiently large $d\in \mathbb N$ if and only if 0 is a lonely point of the corresponding corner cone $C_i$.
(ii) $$\forall \,d\in \mathbb N: {\text {lonely}}_{L}(dS) \supseteq \bigcup \, \{v-de_i\mid \exists \, i>0: v\in {\text {lonely}}_{L}(C_i)\}\cap \mathbb N^m$$.
(iii) The following are equivalent:
  - $$\forall \,i:\#{\text {lonely}}_{L}(C_i)=\infty $$,
  - $$\exists \, i: \#{\text {lonely}}_{L}(C_i)=\infty $$,
  - $$\forall \, r\in \mathbb N\;\exists d\in \mathbb N: \#{\text {lonely}}_{L}(dS)>r$$.

### Proof

(i) Let $d e_i$ be a corner of $dS$, and suppose *d* is large. “$\Rightarrow$” We show: if 0 is not a lonely point of the corner cone $C_i=[e_0-e_i,\dots ,e_m-e_i]$, then $d e_i$ is not a lonely point of *dS*. If 0 is not a lonely point of the corner cone, the corner cone contains some nonzero element of $L$, say $\ell =\alpha _0(e_0-e_i)+\cdots +\alpha _m(e_m-e_i)\in L$ for certain $\alpha _0,\dots ,\alpha _m\in \mathbb N$. Assuming, as we may, that $d>\alpha _0+\cdots +\alpha _m$, we have that $d e_i+\ell$ is an interior point of $dS$ which is equivalent to $d e_i$, proving that $d e_i$ is not lonely. “$\Leftarrow$” We show: if $d e_i$ is not a lonely point of *dS*, then 0 is not a lonely point of the corner cone. Indeed, suppose that $d e_i$ is equivalent to another point *v* of $dS$, say to $v=\beta _1e_1+\cdots +\beta _me_m$ for some $\beta _1,\dots ,\beta _m\ge 0$ whose sum is at most *d*. Then $v-de_i=\beta _1(e_1-e_i)+\cdots +\beta _m(e_m-e_i)+(d-\sum _j\beta _j)(e_0-e_i)$ belongs to the *i*th corner cone, so 0 is not a lonely point of that cone.
(ii) Denote the set on the right hand side by $A_d$. Then $A_d\subset {\text {lonely}}_{L}(dS)$ holds for any *d*: If $v-de_i\in \mathbb N^m$ is such that *v* is lonely in $C_i$, then by Lemma 3.1, $v-de_i$ is lonely in $C_i-d e_i$, which contains $dS$.
(iii) “(a) $\Rightarrow$ (b)” is trivial. “(b) $\Rightarrow$ (c)” is an immediate consequence of part (ii). “(c) $\Rightarrow$ (a)” Suppose that ${\text {lonely}}_{L}(C_i)$ only contains finitely many elements for some corner cone $C_i=[c_1,\dots ,c_n]$. Then, by of Proposition 4.1 there exists a $d'$ such that for every edge $[c_j]$ in $C_i$ the point $d'c_j$ is not lonely. For each such edge we let $d_{j}$ be the minimal euclidean distance of $d'c_j$ to some other element in $C_i$ equivalent to $d'c_j$. Then any point $v=\sum \alpha _jc_j$ in $C_i$ is equivalent to some point in distance $d_{j}$ if $\alpha _j\ge d'$ for some *j*. Setting *d* to be the maximum of the $d_{j}$ this means that every such *v* is equivalent to some point in distance $\le d$. Then a point *v* in $\tilde{d}S$ for $\tilde{d}\ge d$ is lonely only if the coordinates of $v-\tilde{d}e_i$ with respect to the generators $c_j$ of the *i*th corner cone are bounded by *d*, leaving only finitely many possible values for $v-\tilde{d}e_i$.

$$\square $$

For a specific $d\in \mathbb N$, there are only finitely many points in $dS$, and for each of them, we can decide whether it is lonely in a similar way as described above for a given point in a cone. The issue reduces to an integer linear programming question. What we are interested in is how far the number of lonely points can grow as *d* increases. Proposition 4.2 says that the lonely points in $dS$ for sufficiently large *d* are essentially the lonely points of the corner cones. When a cone has only finitely many lonely points, they are all clustered near the apex, so as soon as *d* is sufficiently large, the number of lonely points in the dilated simplex $dS$ is exactly the sum of the number of lonely points in its corner cones. When at least one corner cone has infinitely many lonely points, then the number of lonely points in $dS$ is unbounded as *d* goes to infinity. In summary, we obtain the following algorithm. 
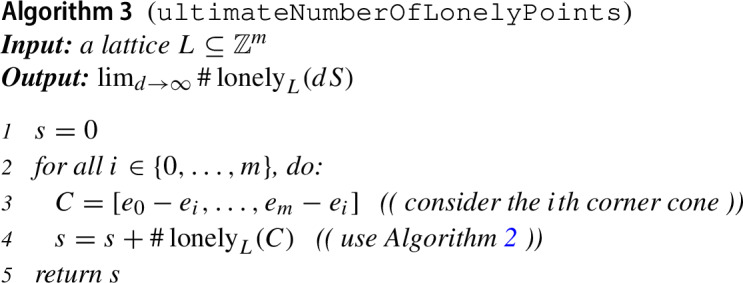


The algorithms described in this section are easy to implement. A proof of concept implementation in Mathematica consists of less than 100 lines of code. This code is available at http://www.kauers.de/software/loneley.m. It is not designed to be efficient but merely meant for the sake of illustration.

## Lonely Points for Small Lattices

It is clear that all integer points in *dS* are lonely when $L=\{0\}$ and that there are no lonely points when $L=\mathbb Z^m$. More generally, geometric intuition suggests that there should be more lonely points when $L$ is “small”. The main result of the present section quantifies this intuition. We show that whenever the dimension of $L$ is less than a certain constant multiple of the ambient dimension *m*, then there is a corner cone which satisfies the conditions of part  of Proposition 4.1 and thus has infinitely many lonely points.

In the subsequent proofs we make use of *sign vectors* and *sign equations*. The possible components of a sign vector are $+$, −, $\oplus$, $\ominus$, or 0. We can assign a sign vector *s* to a given $v\in \mathbb R^m$ in the following way. If the *i*th component of *v* is nonnegative, then the *i*th component of *s* is $+$ or $\oplus$. If the *i*th component of *v* is nonpositive, then the *i*th component of *s* is − or $\ominus$. If a component of *v* is zero, then the corresponding component of *s* can be 0, $+$, −, $\oplus$, or $\ominus$. A component of *s* is $\oplus$ or $\ominus$ only if the absolute value of the corresponding component of *v* is greater than or equal to the sum of the absolute values of all other components. With these rules, any equation $s_1+\dots +s_k=s$ of sign vectors $s_1,\dots ,s_{k},s$ is a *valid sign equation* if there are vectors $v_1,\dots ,v_{k},v\in \mathbb R^m$, such that $v_1+\dots +v_k=v$ and for each $i=1,\dots ,k$, $s_i$ is a *valid sign vector* for $v_i$ and *s* is a valid sign vector for *v*.

### Example 5.1

For the equation

$$\begin{aligned} \begin{pmatrix}-2 \\ 1 \\ 0\end{pmatrix}+\begin{pmatrix}0 \\ -1 \\ 0\end{pmatrix}+\begin{pmatrix}1 \\ 1 \\ -1\end{pmatrix}=\begin{pmatrix}-1 \\ 1 \\ -1\end{pmatrix}, \end{aligned}$$

two valid sign equations are

$$\begin{aligned} \begin{pmatrix}\ominus \\ + \\ -\end{pmatrix}+\begin{pmatrix}+ \\ \ominus \\ -\end{pmatrix}+\begin{pmatrix}+ \\ + \\ -\end{pmatrix}=\begin{pmatrix}- \\ + \\ -\end{pmatrix}\text { and }\begin{pmatrix}- \\ + \\ 0\end{pmatrix}+\begin{pmatrix}0 \\ - \\ 0\end{pmatrix}+\begin{pmatrix}+ \\ + \\ -\end{pmatrix}=\begin{pmatrix}- \\ + \\ -\end{pmatrix}. \end{aligned}$$

We use a shorthand matrix notation

$$\begin{aligned} \begin{bmatrix}s_1&s_2&\ldots&s_k\end{bmatrix}=s \end{aligned}$$

for the sign equation $s_1+\dots +s_k=s$, with the square brackets indicating that the columns of the matrix are summed up to obtain the right hand side. To further shorten notation, we use $\boxed {\oplus }$ and $\boxed {\ominus }$ for nonempty square blocks of the formrespectively, where the number of rows/columns is either clear from the context or irrelevant. Similarly we use , , and  for blocks that only contain $+$, −, or 0 respectively, with the difference that these blocks do neither have to be square blocks nor nonempty.

### Example 5.2

The first sign equation in Example 5.1 can be written as

For any vector *v* in $\mathbb R^m$ we define the *balance* $\tau (v)$ of *v* to be the sum of the components of *v*. The balance of a vector *v* with only nonnegative components is equal to $\Vert v\Vert _1$. For any slanted edge [*c*] of a corner cone, *c* is the difference of two unit vectors, and thus $\tau (c)=0$. For straight edges we have $\tau (c)=\tau (\pm e_i)=\pm 1$.

### Definition 5.1

(visible vectors)  We call a vector $v\in \mathbb R^m$ *i-visible*, if

$$\begin{aligned} (+,\dots ,+,\ominus ,+,\dots ,+) \end{aligned}$$

is a valid sign vector for *v*, where $\ominus$ is at the *i*th position.

The definition is motivated by corner cones. For $i>0$, a vector is *i*-visible if and only if it belongs to $C_i$. An *i*-visible vector *v* has nonpositive balance $\tau (v)\le 0$.

### Lemma 5.1

Let $k\in \{1,\dots ,m\}$ and let $v_1,\dots ,v_k,v\in \mathbb R^m$ be such that $v_1+v_2+\dots +v_k=v$ and that each $v_i$ lies in some corner cone. Suppose that there is an associated sign equation and indices $r_1,\dots ,r_k$ such that a valid sign equation projected to rows $r_1,\dots ,r_k$ is of the formThen for every $j\in \{r_1,\dots ,r_k\}$, the *j*th component of *v* is zero, and for every $j\in \{1,\dots ,m\}\setminus \{r_1,\dots ,r_k\}$, the *j*th component of $v_i$ is zero for every *i*.

### Proof

Let $\pi :\mathbb R^m\rightarrow \mathbb R^{k}$ be the projection on the components with indices $r_1,\dots ,r_k$, and $\overline{\pi }$ the projection on the complementary components. The sign equation implies that $\tau (\pi (v_i))\le 0$ for all $v_i$. It follows that $\tau (\pi (v))$ has to be less than or equal to 0 as well. As $\pi (v)$ only contains nonnegative entries, this is only possible if $\pi (v)$ is the zero vector. This shows the first part and also implies the equation

$$\begin{aligned} \tau (\pi (v_1)) + \tau (\pi (v_2)) + \dots + \tau (\pi (v_k))=0. \end{aligned}$$

Since no summand on the left hand side is strictly positive, all the $\tau (\pi (v_i))$ have to be equal to 0. As every $v_i$ lies in some corner cone, and their negative components only have indices contained in $\{r_1,\dots ,r_k\}$, we get that all the $\overline{\pi }(v_i)$ only have nonpositive components. Now it follows that all $\overline{\pi }(v_i)$ are equal to zero, since

$$\begin{aligned} 0\ge \tau (\overline{\pi }(v_i))=\tau (\overline{\pi }(v_i))+\tau (\pi (v_i))=\tau (v_i)\ge 0. \end{aligned}$$

$$\square $$

### Remark 5.1

Clearly, if a $v_1,\dots ,v_k,v$ with $v_1+\dots +v_k=v$ are such that a valid sign equation contains rows of the formthen *v* and the $v_i$ can only contain zero entries at the corresponding indices.

### Proposition 5.1

Let $v_1,\dots ,v_m\in \mathbb R^m\setminus \{0\}$ be such that each $v_i$ is *i*-visible. If no subspace of $V:=\mathbb R v_1+\dots +\mathbb R v_m$ can be decomposed into a direct sum of more than one nonzero vector spaces, then $\dim (V)= m-1$.

### Proof

If *V* is of dimension *m*, it can be decomposed into the direct sum of *m* nonzero vector spaces. Suppose that $\dim (V)<m-1$, and, without loss of generality, that $v_1,\dots ,v_{m-2}$ generate *V*, i.e., there are $\alpha _1,\dots ,\alpha _{m-2}$ and $\beta _1,\dots ,\beta _{m-2}\in \mathbb R$ with $v_{m-1}=\sum _{i<m-1} \alpha _iv_i$ and $v_m=\sum _{i<m-1}\beta _iv_i$. For these we get corresponding sign equations

2 $$\begin{aligned} \pm \begin{pmatrix} \ominus \\ +\\ \vdots \\ +\\ +\\ +\\ +\end{pmatrix} \pm \dots \pm \begin{pmatrix} +\\ +\\ \vdots \\ +\\ \ominus \\ +\\ +\end{pmatrix} = \begin{pmatrix} +\\ +\\ \vdots \\ +\\ +\\ \ominus \\ + \end{pmatrix}, \end{aligned}$$

3 $$\begin{aligned} \pm \begin{pmatrix} \ominus \\ +\\ \vdots \\ +\\ +\\ +\\ +\end{pmatrix} \pm \dots \pm \begin{pmatrix} +\\ +\\ \vdots \\ +\\ \ominus \\ +\\ +\end{pmatrix}=\begin{pmatrix} +\\ +\\ \vdots \\ +\\ +\\ +\\ \ominus \end{pmatrix}, \end{aligned}$$

where the ± reflect the fact that the $\alpha _i$ and $\beta _i$ can be positive or negative. We show that there is no combination of signs for the $\alpha _i$ and $\beta _i$ such that both equalities hold, unless *V* can be decomposed into a direct sum. We first look at (3). From the last row we see that at least one $\beta _i$ has to be strictly negative, as no $v_i$ on the left hand side has a negative entry at index *m*, and $v_{m}$ is nonzero. So we can split the vectors into two groups: those with positive $\beta _i$ and those with strictly negative $\beta _i$. After changing the summation order and reorganizing the rows if necessary, (3) becomesSuppose at least one $\beta _i$ is strictly positive. Then Lemma 5.1 implies that some components have to be zero, and we get a block diagonal formThus, the $v_i$ appearing with a nonzero coefficient in (2) span a vector space that can be decomposed into a direct sum if at least one $\beta _i$ is strictly positive. Otherwise, with the analogous reasoning for $v_{m-1}$, we can suppose that all the $\alpha _i$ and $\beta _i$ are nonpositive, and conclude that the sign equations for $v_{m-1}$ and $v_m$ are of the form45If all $\alpha _i$ (implicitly used in (4)) were nonzero, then the last row in (4) implies that the last components of all the $v_i$ would have to be zero, which is incompatible with the last row in (5). Again with the analogous reasoning for the $\beta _i$ we see that not all $\alpha _i$ and not all $\beta _i$ are nonzero. As before we split the vectors on the left hand side of each equation into two blocks: vectors that appear with a nonzero coefficient in only one of the equations and vectors that are shared in both equations with nonzero coefficients, which gives, after reordering the rows and summands if necessary:We use Remark 5.1 to determine zero components in the first equation:Then, doing the same for the second equation, and using the fact that we already know some zero components in the shared vectors, we get:Denote the number of shared vectors by *s*. If *s* is greater than 0, we look at the rows in the equation for $v_{m-1}$ where the shared vectors are nonzero:As all nonshared vectors on the left hand side only have negative components, we can bring them to the right hand side and get:Note that here, all the hidden entries of the shared vectors are zero. We can suppose that the shared vectors are linearly independent, otherwise we could replace some coefficients with zero. As they are linearly independent, however, they span the whole space $\mathbb R^s$, thus the shared vectors can be replaced by unit vectors, which leads to a decomposition of *V* into a direct sum of vector spaces. It remains to handle the case where there are no shared vectors in (4) and (5). In that case, certain components in (4) and (5) have to be zero:The zero entries on the left hand side imply that the space spanned by *V* can be decomposed into a direct sum. This completes the proof. $\square$

### Corollary 5.1

Let $L\subseteq \mathbb Z^m$, $m\ge 3$, be a lattice of dimension less than $m-1$ such that no subspace of the vector space spanned by $L$ can be decomposed into a direct sum of two nonzero spaces. Then there exists a corner cone $C$ of the standard simplex such that $L\cap C=\{0\}$.

### Proof

Let $v_i\in L\cap C_i$ for all $i=1,\dots ,m$ and assume they are all nonzero. Then, since each $v_i$ is *i*-visible, Proposition 5.1 yields

$$\begin{aligned} \dim (L)\ge \dim {\langle v_1,\dots ,v_m\rangle }=m-1, \end{aligned}$$

a contradiction. $$\square $$

### Corollary 5.2

Let $L=\langle v_1,\ldots ,v_k\rangle$ be a lattice in $\mathbb Z^m$, $m\ge 3$. If $k=\dim (L)<{2}m/3$, then there exists a corner cone $C$ of the standard simplex such that $L\cap C=\{0\}$.

### Proof

Using Corollary 5.1 and projecting to the relevant coordinates shows that any subset *S* of $v_1,\dots ,v_k$ of some cardinality *s* such that *S* cannot be decomposed into a direct sum can only contain nonzero vectors of $s+1$ corner cones. Additionally for $s=1$, *S* can only contain nonzero vectors of one corner cone. In fact, if there is a $v\in \{v_1,\dots ,v_k\}$ and an $i\in \{1,\dots ,m\}$ such that *v* is *i*-visible, then it is immediate from the sign vector $(+,\dots ,+,\ominus ,+,\dots ,+)$ of *v* that $\langle v\rangle \cap C_j=\{0\}$ for all $j\ne i$. It follows that *V* can be decomposed into the sum of at most *k*/2 many two-dimensional vector spaces, each containing nonzero vectors of three corner cones. $\square$

**Fig. 4:**
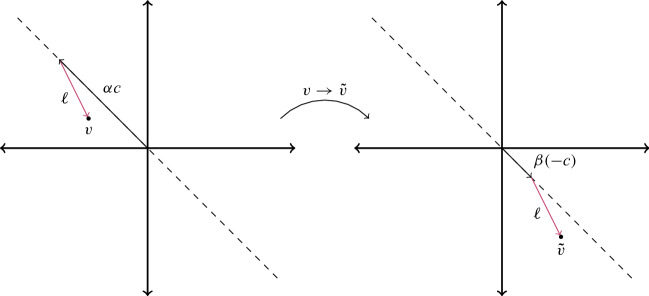
Illustration of Lemma 5.2 in dimension 2 with $c=(-1,1)$, $\ell =(1,-2)$, $v=(-2,1)$, $\alpha =3$, $\gamma =4$, $\beta =1$, and $\tilde{v}= (2,-3)$

In order to derive a dimension bound such that both conditions in part  of Proposition 4.1 are met, we need the following lemma that allows us to construct a nonlonely point in a corner cone from a nonlonely point in a different corner cone. A geometric interpretation of the statement is given in Fig. 4.

### Lemma 5.2

Let $C_i\subset \mathbb Z^m$ be a corner cone, [*c*] be a slanted edge in $C_i$, and let $j\in \mathbb N$ be such that the *j*th component of *c* is 1. If $\ell \in \mathbb Z^m$ and $\alpha \in \mathbb N$ are such that $v:=\ell + \alpha c\in C_i$, then there exists a $\beta \in \mathbb N^*$ with $\ell +\beta (-c)\in C_j\setminus \{0\}$, where $[-c]$ is a slanted edge in the corner cone $C_j$.

### Proof

By definition, the components of *c* are all zero except for the *i*th component, which is $-1$, and the *j*th component for some $j\ne i$, which is 1. Thus $[-c]$ is a slanted edge in $C_j$. Set $\gamma :=\max (-v_i,\alpha )+1$, where $v_i$ is the *i*th component of *v*. Then $\tilde{v}:=v-\gamma c$ is *j*-visible, as $\tilde{v}_i = v_i+\gamma >0$, $\tilde{v}_k=v_k\ge 0$ for all $k\ne i,j$ and $\tilde{v}_j = v_j - \gamma \le 0$ with

$$\begin{aligned} -\tilde{v}_j = -v_j+\gamma = -v_j-v_i+(v_i+\gamma ) >-v_j + \sum _{k\ne i} v_k+ \tilde{v}_i=\sum _{k\ne j}\tilde{v}_k. \end{aligned}$$

Then, with $\beta :=\gamma -\alpha \in \mathbb N^*$, we get $\ell + \beta (-c) = \ell +(\alpha -\gamma )c = \tilde{v}\in C_j$. $\square$

### Theorem 5.1

Let $L$ be a lattice in $\mathbb Z^m$. If $\dim (L)<(m-4)/3$, then there exists a slanted edge [*c*] in a corner cone $C$ such that all elements of [*c*] are lonely.

### Proof

If $m\le 4$, there is nothing to show. Suppose $m>4$. Without loss of generality, we may assume *m* is even, because if it is odd, then both $(m-4)/3$ and $((m+1){-}4)/3$ are fractions, so we have $\dim (L)<(m-4)/3$ iff $\dim (L)<((m+1)-4)/3$, so we may add without harm an extra dimension to the setting.

By of Proposition 4.1, we have to show that $L\cap C =\{0\}$ and $(L+\langle c\rangle )\cap C=[c]$. If there exist such *c* and $C$, then there is no nonzero $\ell \in L$ and no nonzero $\alpha \in \mathbb Z$ such that $\ell +\alpha c\in C$. Thus we can prove the claim by showing that if for every corner cone $C_i$, $i=1,\dots ,m$, and every slanted edge $[c_{i,j}]$ of $C_i$, $j=1,\dots ,m$, $j\ne i$, there are a nonzero $\ell _{i,j}\in \mathbb Z^m$ and a nonzero $\alpha _{i,j}\in \mathbb Z$ such that the vector $v_{i,j}:=\ell _{i,j}+\alpha _{i,j}c_{i,j}$ is *i*-visible, then $\dim (L)\ge (m-4)/3$. So suppose such $\ell _{i,j}$ and $\alpha _{i,j}$ exist. Then  is a valid sign vector for $v_{i,j}$, with $\ominus$ at the *i*th position. We first show that each $\ell _{i,j}$ is either *i*-visible, *j*-visible, or has exactly two strictly negative entries, at indices *i* and *j*. For the moment, we focus on $i=1$, $j=2$, allowing us to drop both indices. The reasoning for all other pairs *i*, *j* is analogous. We get the equation $\ell + \alpha c = v$. If $\alpha \le 0$, we can add $-\alpha c$ to both sides of the equation, not perturbing the 1-visibility of the right hand side, which shows that $\ell$ is 1-visible. Otherwise, we get a sign equation with unknown entries for $\ell$,The signs for all but two components of $\ell$ are immediate:As $\tau (c)=0$ and $\tau (v)\le 0$, we get that $\tau (\ell )\le 0$. Thus, if the second component of $\ell$ is positive, then it follows that $\ell$ is 1-visible with a strictly negative first component. If the second component of $\ell$ is negative, we can apply Lemma 5.2 to see that there exists a $\beta \in \mathbb N^*$ such that $\ell +\beta (-c)$ is 2-visible, yieldingWith the same reasoning as above we can determine that $\ell$ is either 2-visible or its first component is strictly negative. This shows our claim for the $\ell _{i,j}$. It follows that for each pair (*i*, *j*), the vector $\ell _{i,j}$ is such that it has a strictly negative entry at *i*, or *j*, or both. Thus we can find at least *m*/2 pairwise different $\ell _1,\ldots ,\ell _{m/2}\in L$ such that no two $\ell _i$ have a negative entry at the same index, and for each index in $\{1,\dots ,m\}$, there is exactly one $\ell _i$ with a negative entry at that position. We now map these lattice elements to *i*-visible vectors, $i=1,\ldots ,m/2-2$, in $\mathbb Z^{m/2}$.

For any permutation $\pi$ of $1,\ldots ,m$ consider the surjective linear mapThere are $n(m):=m!/2^{m/2}$ many such maps. We say a vector *u* and a map $\psi _\pi$ are compatible, if:

- *u* is *i*-visible for some *i*, and $\psi _\pi (u)\ne 0$. If $a\in \mathbb N$ is such that $\pi (a)=i$, then $\psi _\pi$ is $\lfloor (a+1)/2\rfloor$-visible.
- *u* contains exactly two strictly negative entries at indices *i* and *j*, and there is an odd integer *a* such that $\pi (a)=i$ and $\pi (a+1)=j$, i.e., when applying $\psi _\pi$ on *v*, the two negative entries are added together to give an $((a+1)/2)$-visible vector.

We now show that there exists a permutation $\pi$ such that at least $m/2-2$ many $\ell _i$ are compatible to $\psi _\pi$. In fact we can choose $\pi$ such that all $\ell _i$ with exactly two negative entries are compatible with $\psi _\pi$, as they do not have negative entries at the same indices. This leaves us with some even number $k\ge 0$ of indices not yet considered for $\pi$ and *k* many $\ell _i$ that could potentially be incompatible to such a permutation. Furthermore, there are *n*(*k*) many permutations left to choose from. Each of the remaining $\ell _i$ is contained in a different corner cone, say $C_i$, and so $\ell _i$ is incompatible if $\psi _\pi (\ell _i)=0$. For $k> 2$, each $\ell _i$ can be in the kernel of at most $n(k-2)$ many of the remaining permutations (this is the case if $\ell _i$ is contained in a slanted edge of a corner cone). As there are *k* ($k>2$, even) many such $\ell _{i}$, there has to be a $\psi _\pi$ for which the number of *i*-visible $\ell _i$ that are mapped to zero is at most

$$\begin{aligned} \biggl \lfloor k\frac{n(k-2)}{n(k)}\biggr \rfloor =\biggl \lfloor \frac{2}{k-1}\biggr \rfloor =0. \end{aligned}$$

For $k=2$, there is only one choice for $\pi$, and we could be in the situation where both of the $\ell _i$ have to be mapped to zero. For any such $\pi$, the images of the $\ell _{i}$ therefore contain at least $m/2-2$ many nonzero vectors with $m/2-2$ different sign patterns (after potentially reordering the rows)

$$\begin{aligned} \begin{pmatrix}\ominus \\ +\\ \vdots \\ +\\ +\\ +\end{pmatrix},\begin{pmatrix}+\\ \ominus \\ \vdots \\ +\\ +\\ +\end{pmatrix},\dots ,\begin{pmatrix}+\\ +\\ \vdots \\ \ominus \\ +\\ + \end{pmatrix}. \end{aligned}$$

By projecting to the first $m/2 -2$ coordinates and using Corollary 5.2, it follows that

$$\begin{aligned} \dim {\langle \ell _{1},\ldots ,\ell _{m/2}\rangle }=\dim {\langle \psi _\pi (\ell _{1}),\ldots ,\psi _\pi (\ell _{m/2})\rangle }\ge \frac{2}{3}\biggl (\frac{m}{2}-2\biggr )=\frac{m-4}{3}. \end{aligned}$$

This proves the claim.$\square$

Without further restrictions on $L$, there is no analogous result for straight edges, i.e., there is no upper bound for the dimension proportional to *m* such that lower dimensional lattices necessarily lead to infinitely many lonely points on at least one straight edge. For any *m*, the lattice generated by $(1,0,\dots ,0)$ yields only finitely many lonely points on any straight edge.

## Conclusion and Open Questions

We translated the problem of reducing the order of a C-finite sequence to questions about which points in a dilated simplex are not connected to any other point in the simplex via a specific lattice. Our answers to these questions are in the form of algorithms that determine when the number of these points grows indefinitely with the dilation, and also compute the exact number if there are only finitely many lonely points. Furthermore we showed that if the dimension of the lattice is small enough, then the number of lonely points always grows indefinitely.

Theorem 5.1 is helpful for our original application to C-finite sequences, because the lattices appearing in this context are typically small, often even empty. We do not know however whether the bound of Theorem 5.1 is tight enough to cover all cases of interest. If it is not, we can still use the algorithms from Sect. 4 to see whether there are enough lonely points to derive a finite degree bound for the ansatz.

### Example 6.1

The Perrin numbers $(P_n)_{n=0}^\infty$ are defined by $P_{n+3}=P_n+P_{n+1}$ and $P_0=3$, $P_1=0$, $P_2=2$. Using the results of this paper, we an show that there is no polynomial *p*(*x*) such that the C-finite sequence $(p(P_n))_{n=0}^\infty$ satisfies a recurrence of lower order. Indeed, the exponent lattice in this example is generated by (1, 1, 1), and Algorithm 1 applied to this lattice asserts that there are infinitely many lonely points. This means that all the points of at least one edge of a dilated simplex *dS* are lonely. This in turn means that in step 3 of the algorithm from Sect. 2, we have $\ell \ge d$, and since we need $\ell \le 4$ in order to ensure that in step 4 we have at least one *I* of size 2 that does not contain a lonely point, we get the degree bound $d=4$. As the algorithm returns “no solution” for $d=4$, we can conclude that there does not exist a polynomial *p* of any degree such that $(p(P_n))_{n=0}^\infty$ satisfies a recurrence of order 2.

As for extensions of our theoretical results, there are immediate questions that are rooted in discrete geometry: Can we find a closed form expression depending on *d* for the number of lonely points in $dS$ for a given lattice? How many lonely points are there in more involved convex polytopes? How do linear transformations on the lattice affect lonely points? Although questions of this kind are not directly related to our initial number theoretic motivation, their pursuit may still lead to valuable insight.

## Footnotes

1 W.l.o.g., we consider $\mathbb C$ instead of an algebraically closed arbitrary field of characteristic zero.
